# Supplementary material for: Co-Spray Dried Nafamostat Mesylate with Lecithin and Mannitol as Respirable Microparticles for Targeted Pulmonary Delivery: Pharmacokinetics and Lung Distribution in Rats
Source: Pharmaceutics. 2021 Sep 19;13(9):1519. doi: 10.3390/pharmaceutics13091519 (PMC8468663; doi:10.3390/pharmaceutics13091519)
Supplement: Supplementary file 1 [file pharmaceutics-13-01519-s001.zip › pharmaceutics-1374892-supplementary.pdf]

pharmaceutics

MDPI

Supplementary Materials: Co-Spray Dried Nafamostat Mesylate with Lecithin and Mannitol as Respirable Microparticles for Targeted Pulmonary Delivery: Pharmacokinetics and Lung Distribution in Rats

Ji-Hyun Kang, Young-Jin Kim, Min-Seok Yang, Dae Hwan Shin, Dong-Wook Kim, Il Yeong Park and Chun-Woong Park

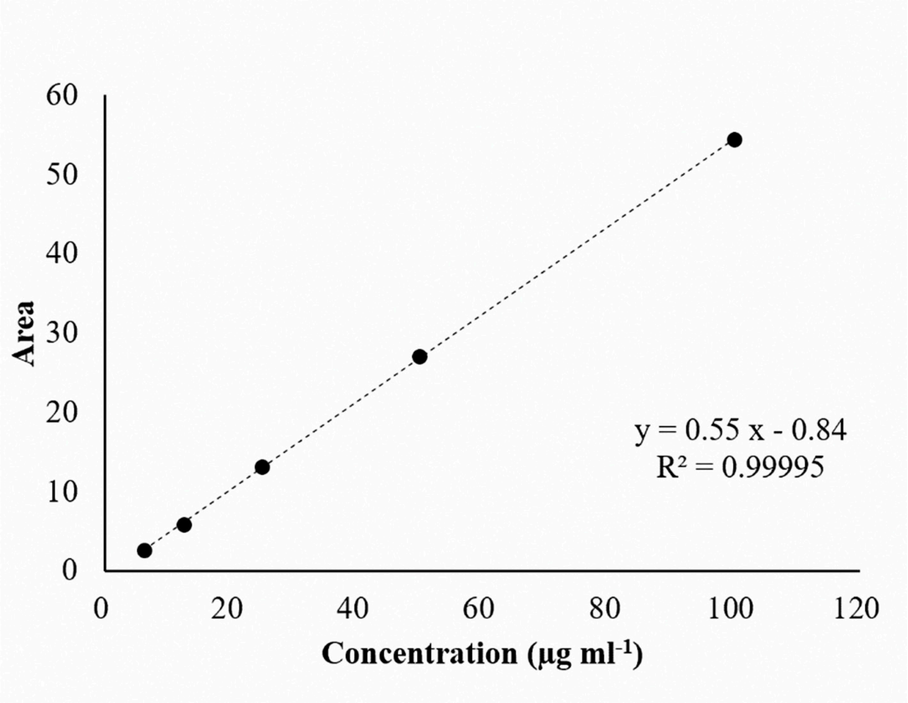

Figure S1. Linearity of nafamostat mesylate (NFM) HPLC analysis method validation.

Table S1. Linearity of nafamostat mesylate (NFM) HPLC analysis method validation.

Concentration (μg mL^{-1})	Area	Calculated Concentration (μg mL^{-1})	Deviation (%)
6.25	2.58	6.18	1.10
12.50	5.897	12.17	2.64
25.00	13.182	25.32	1.29
50.00	26.975	50.22	0.45
100.00	54.465	99.85	0.15
**R^2**	0.99995		
**Slope**	0.55		
**Intercept**	−0.84		

Table S2. Accuracy of nafamostat mesylate (NFM) HPLC analysis method validation.

Sample No.	Concentration (μg mL^{-1})	area	Founded Concentration (μg mL^{-1})	recovery (%)
**1**	25.00	13.75	26.34	105.37
**2**	50.00	27.52	51.20	102.40
**3**	100.00	55.29	101.34	101.34
**4**	25.00	13.80	26.44	105.75
**5**	50.00	27.58	51.31	102.62
**6**	100.00	55.06	100.93	100.93
**7**	25.00	13.88	26.59	106.35
**8**	50.00	27.53	51.23	102.46
**9**	100.00	55.16	101.10	101.10
			**Average**	103.15
			**Standard deviation**	2.11
			**%RSD**	2.05

Table S3. Precision of nafamostat mesylate (NFM) HPLC analysis method validation.

Sample No.	Concentration (μg mL^{-1})	area
**1**	100	54.26
**2**	100	54.10
**3**	100	54.28
**4**	100	54.94
**5**	100	54.41
**6**	100	55.09
	**Average**	54.52
	**Standard deviation**	0.40
	**%RSD**	0.74
